# Dietary nitrate ingested with and without pomegranate supplementation does not improve resistance exercise performance

**DOI:** 10.3389/fnut.2023.1217192

**Published:** 2023-07-06

**Authors:** Rachel Tan, Katherine M. Price, Lauren E. Wideen, Isabella G. Lincoln, Sean T. Karl, Jacob P. Seals, Keonabelle K. Paniagua, Dylan W. Hagen, Isaac Tchaprazian, Stephen J. Bailey, Adam Pennell

**Affiliations:** ^1^Department of Sports Medicine, Pepperdine University, Malibu, CA, United States; ^2^School of Sport, Exercise and Health Sciences, Loughborough University, Loughborough, United Kingdom

**Keywords:** nitric oxide, power, strength training, beetroot, neuromuscular, muscle, weightlifting, exercise

## Abstract

This study tested the hypothesis that co-ingesting nitrate (NO_3_^−^)-rich beetroot juice (BR) and pomegranate powder (POM) would enhance neuromuscular performance during vertical countermovement jumps, explosive kneeling countermovement push-ups, and back squats compared to BR ingestion alone. Fifteen recreationally-active males were assigned in a double-blind, randomized, crossover design, to supplement in 3 conditions: (1) NO_3_^−^-depleted beetroot juice (PL; 0.10 mmol NO_3_^−^) with two empty gelatin capsules; (2) NO_3_^−^-rich beetroot juice (BR; 11.8 mmol NO_3_^−^) with two empty gelatin capsules, and (3) BR with 1,000 mg of POM powder in two capsules (BR + POM). Participants completed 5 countermovement jumps and 5 kneeling countermovement push-ups interspersed by 1  min of recovery. Subsequently, participants performed 2 sets of 2 × 70% one-repetition maximum back squats, interspersed by 2  min of recovery. Plasma [NO_3_^−^] and nitrite ([NO_2_^−^]) were elevated following BR and BR + POM compared with PL and POM (*p* < 0.001) with no differences between BR and BR + POM (*p* > 0.05) or PL and POM (*p* > 0.05). Peak power during countermovement jumps increased by 3% following BR compared to BR + POM (88.50 ± 11.46 vs. 85.80 ± 10.14 W/Kg^0.67^, *p* = 0.009) but not PL (88.50 ± 11.46 vs. 85.58 ± 10.05 W/Kg^0.67^, *p* = 0.07). Neuromuscular performance was not different between conditions during explosive kneeling push-ups and back squats (*p* > 0.05). These data provide insight into the efficacy of NO_3_^−^ to modulate explosive resistance exercise performance and indicate that supplementing with BR alone or combined with POM has limited ergogenic potential on resistance exercise. Furthermore, caution is required when combining BR with POM, as this could compromise aspects of resistance exercise performance, at least when compared to BR ingested independently.

## 1. Introduction

Dietary NO_3_^−^ supplementation can increase endogenous nitric oxide (NO), a regulatory molecule involved in a plethora of physiological functions (1), via the conversion of NO_3_^−^ to nitrite (NO_2_^−^) and then NO_2_^−^ to NO (2). While initial studies indicated enhanced performance during continuous submaximal endurance exercise after NO_3_^−^ supplementation (3), emerging evidence indicates the potential of dietary NO_3_^−^ supplementation to enhance performance during high-power and high-velocity contractions (4). Improved performance during short-duration, high-intensity exercise requiring high-velocity contractions might be linked to preferential effects of NO_3_^−^ supplementation on type II muscle fibers (5, 6). To date, the vast majority of dietary NO_3_^−^ research has revolved around cycling and running exercise (3, 7) with fewer studies evaluating its ergogenic potential in other exercise modalities, such as resistance exercise (8). From the studies conducted to date, NO_3_^−^ has been shown to be effective (9, 10) and ineffective at improving power output during back squats (11). It is possible that the efficacy of NO_3_^−^ on performance is greater in upper body exercises, consequent to a greater proportion of type II muscle fibers in some upper body compared to lower body skeletal muscles (12), but data from studies examining the effects of NO_3_^−^ on power output during bench press exercise are also conflicting (11, 13). Furthermore, the ergogenic potential of NO_3_^−^ on explosive body mass resisted outcomes are scarce. For example, only a few studies have examined the effects of NO_3_^−^ on vertical countermovement jumps and these studies have yielded equivocal effects (14, 15) and to date, no study has examined explosive upper body exercise. Therefore, further research is required to improve our understanding on the ergogenic potential of NO_3_^−^ for resistance-type exercise.

It has been reported that a greater increase in plasma [NO_2_^−^] following NO_3_^−^ ingestion is associated with greater improvements in performance during cycling (16, 17) and knee extensor strength (18) assessments. Accordingly, it is possible that combining increased dietary NO_3_^−^ intake with another supplement with the potential to further increase NO bioavailability could elicit additional performance enhancements compared to NO_3_^−^ ingestion alone (18). Theoretically, antioxidants could increase NO bioavailability by quenching reactive oxygen species (ROS) and attenuating the scavenging of NO by ROS (19). Despite the potential synergistic effect from co-ingesting NO_3_^−^ with other antioxidant compounds on NO bioavailability and NO_3_^−^-induced ergogenic effects, it is also possible that combining NO_3_^−^ and antioxidants could shift skeletal muscle redox balance to an extent that compromises contractile function given that low ‘physiological’ concentrations are required for normal contractile processes (20). Therefore, the extent to which co-ingestion of NO_3_^−^ with other antioxidants impacts NO bioavailability and exercise performance requires further empirical exploration.

Pomegranate is purported to confer antioxidant effects (21) which could augment the synthesis, bioavailability, and physiological effects of NO (22). In addition, pomegranate has been suggested to be a rich source of NO_3_^−^ (23), but to date, only two studies have examined the effects of pomegranate supplementation on NO bioavailability (24, 25). Both studies reported that pomegranate supplementation increased plasma [NO_3_^−^] compared to a placebo condition but the authors did not employ analytical procedures that were sensitive to determine plasma [NO_2_^−^] (24, 25), which is a crucial biomarker of NO bioavailability and associated with the efficacy of NO_3_^−^ supplementation on performance (16). Furthermore, only one study has examined the ergogenic potential of pomegranate supplementation during resistance exercise performance (26). In this study, the authors observed that the maximum load and volume of snatch and clean and jerk were improved following 2  days of pomegranate juice supplementation (26). Given the potential for pomegranate to improve NO bioavailability and resistance exercise performance, co-ingesting NO_3_^−^ with pomegranate could induce synergistic effects but has yet to be explored. In support of the possibility of additional effects when NO_3_^−^ is co-ingested with, compared to without, polyphenols and antioxidants, are data reporting a greater magnitude of increase in plasma [NO_2_^−^] and lowered blood pressure after consuming BR and spinach, compared to an equimolar sodium NO_3_^−^ dose (27). Furthermore, beneficial physiological effects of NO_3_^−^ were observed in cycling following BR but not an equivalent dose of sodium NO_3_^−^ (28). Accordingly, there is rationale to explore the potential synergistic effects of NO_3_^−^ and pomegranate supplementation on resistance exercise performance.

The purpose of this study was to investigate the effects of acute co-ingestion of dietary NO_3_^−^, provided as beetroot juice, and pomegranate powder on plasma [NO_3_^−^] and [NO_2_^−^], and neuromuscular performance during vertical countermovement jumps, kneeling countermovement push-ups, and back squats. We hypothesized that dietary NO_3_^−^ supplementation would enhance physiological and performance variables during vertical countermovement jumps, kneeling countermovement push-ups, and back squats, and that effects would be greater following the co-ingestion of NO_3_^−^ and pomegranate concomitant with elevated plasma [NO_2_^−^], compared to NO_3_^−^ ingestion alone.

## 2. Materials and methods

### 2.1. Participants

Fifteen healthy recreationally active men (mean ± SD: age 21 ± 1 years, body mass 78 ± 13 kg, height 1.78 ± 0.08 m) volunteered to participate in this study following a power calculation based on a published report (13) using a power of 0.95 and alpha of 0.05. All participants were university students and were given a random identification code for anonymization. Recreationally active was defined as individuals who performed resistance exercise at least twice weekly, and individuals were instructed to maintain their normal training regimens throughout the experiment. Participants completed a screening and a physical activity readiness questionnaire. The participant exclusion criteria were individuals with contraindications to exercise, cardiometabolic disease, currently consuming dietary supplements containing caffeine, sodium bicarbonate, creatine, beta-alanine, and/or NO precursor supplements (i.e., NO_3_^−^, arginine, citrulline, antioxidants), females, and smokers. Females were excluded given that sex-differences in the physiological responses to NO_3_^−^ ingestion may exist (29) and that including the appropriate controls (i.e., testing only during the early follicular phase) would have been unfeasible logistically (30). The experimental protocols, risks, and benefits of participating were explained prior to participants providing written informed consent. This study was pre-registered on the Open Science Framework database (osf.io/ekvwz) on 19 January 2023, was approved by the Institutional Research Ethics Committee and conformed to the code of ethics of the Declaration of Helsinki.

### 2.2. Experimental overview

**Figure 1:**
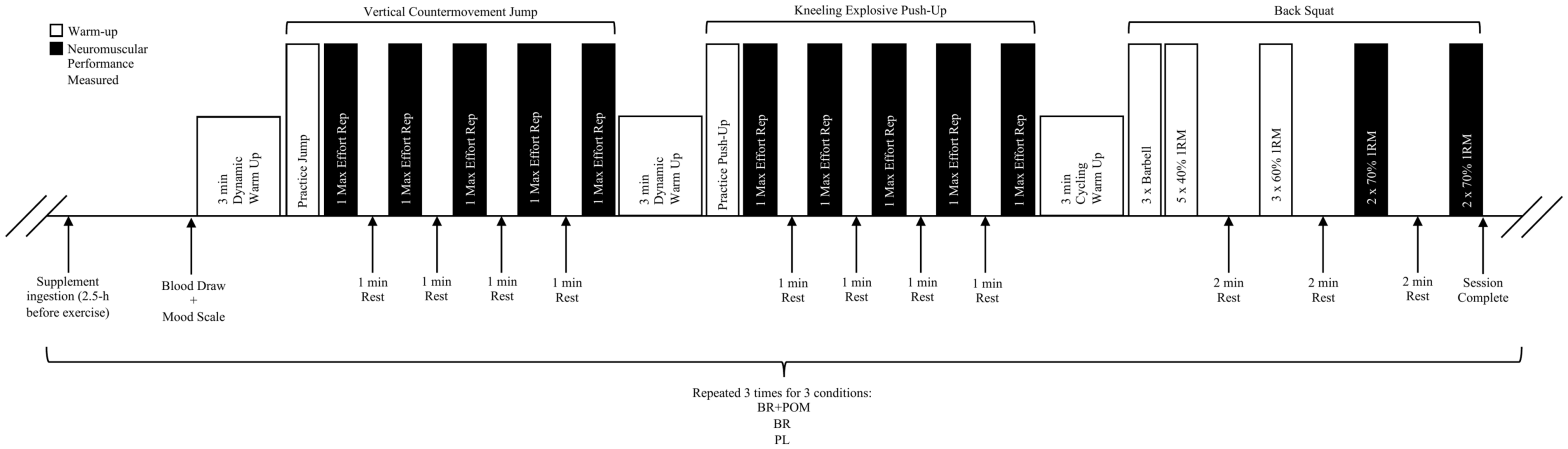
Schematic diagram of exercise protocol.

Participants reported to the laboratory on a total of five occasions over a 4-wk period (Figure 1). During visit 1, participants underwent standardized one-repetition maximum (1RM) testing procedures for the determination of the resistance to be applied in subsequent visits. During visit 2, participants performed a protocol and coaching technique familiarization to ensure correct lifting technique. Subsequently, in a double-blind, randomized, crossover design, participants were assigned to three experimental conditions using a web-based randomizer^1^ to receive acute NO_3_^−^-rich beetroot juice (BR) or NO_3_^−^-depleted beetroot juice (PL), in addition to gelatin capsules that were empty or containing 1,000 mg of pomegranate powder (POM) (23), 2.5 h prior to the commencement of the exercise protocol. All of the supplement bottles and capsules were identical in size, smell, taste and appearance. Each condition was separated by a wash-out period of at least 5 days. Participants recorded their physical activity and diet during the 24 h prior to the first experimental visit and were asked to repeat these for subsequent visits. All tests were performed at the same time of day (±2 h). Prior to their first visit, participants were instructed to avoid antibacterial mouthwash for the duration of the study, given that mouthwash has been evidenced to interfere with NO_3_^−^ metabolism in humans (31). Additionally, subjects were to refrain from strenuous exercise and alcohol 24 h prior to each experimental visit, NO_3_^−^-rich foods (i.e., beetroot, celery, lettuce, radish, spinach etc.) and antioxidant-rich foods 48 h prior to visit, and caffeine 12 h before visit. The lead researcher, data collectors, and participants were blinded to the conditions. The distribution of supplements for each condition was performed by a researcher that was not formally involved in data collection processes, thereby limiting the potential of bias.

### 2.3. Exercise protocols

Participants performed a warm-up in preparation for 1RM testing as previously described (13). Briefly, participants completed 5 back squats at 50% of their perceived 1RM, followed by 3 repetitions at 70% of their perceived 1RM with each set interspersed by 2 min of recovery. Subsequently, the load was increased in stepwise increments (0.2–9 kg) until the participant’s maximum was successfully lifted within 3 to 5 attempts, with each attempt interspersed by 3 min of recovery. All participants were required to use standardized procedures for the back squat (i.e., medium grip, parallel depth, neutral stance, lower body extension to original standing position) throughout the entire duration of the study and were provided coaching techniques.

During visit 2, participants performed a familiarization to the exercise protocol to ensure correct lifting technique and to minimize any potential learning effects. Participants performed a standardized warm-up consisting of dynamic stretching, followed by 5 vertical countermovement jumps, interspersed by 1 min of recovery. After 3 min of recovery, participants performed 5 kneeling countermovement push-ups, interspersed by 1 min of recovery. After 3 min of recovery, participants performed an explosive lift using the barbell only (20 kg) for a total of 3 repetitions. Coaching techniques were provided during this session.

During the experimental visits (i.e., visits 3, 4, 5), participants reported to the laboratory to perform the experimental protocol to determine muscular power, and explosive performance as familiarized with on visit 2. A resting venous blood sample was obtained at rest before the commencement of exercise. The movement tempo of individual movement phases during resistance exercise were controlled for using an eccentric-pause-concentric-pause tempo of 1–0–1-2 to emphasize explosive movements and to standardize lifting across participants (32). To optimize the preservation of exercise intensity, smaller muscle groups as well as fatigue-inducing exercises were performed later within the exercise protocol (e.g., lower body before upper body; unweighted low-rep ballistic exercises before loaded lifts) (33). During these visits, participants performed a standardized warm-up followed by 5 maximal vertical countermovement jumps, interspersed by 1 min of recovery. After 3 min of recovery, participants performed 5 kneeling countermovement push-ups, interspersed by 1 min of recovery. Subsequently, participants completed an unweighted cycling warm-up at 60 rpm (Monark 828E, Monark Sports and Medical, Sweden) for 3 min, then performed a warm-up for the back squat as previously described (13) consisting of 3 repetitions with the barbell only, followed by 5 repetitions at 40% 1RM, followed by 3 repetitions at 60% 1RM, with each set interspersed by 2 min of recovery. Following this, a linear position transducer (GymAware, Kinetic Performance Technology, Mitchell, Australia) was attached to the barbell to assess power and velocity of movement. Power and velocity were determined in a protocol consisting of 2 sets x 2 repetitions at 70% 1RM with each set interspersed by 2 min of recovery. Participants were instructed to lift the weight as fast as possible, and encouragement and technical feedback was given to participants during all sets.

### 2.4. Supplementation procedures

Participants were randomly assigned to three experimental conditions to consume: (1) NO_3_^−^-depleted beetroot juice (PL: 0.10 mmol of NO_3_^−^ per 70 mL, Beet it; James White Drinks, Ipswich, UK) with two empty gelatin capsules (Bulk Supplements, Hard Eight Nutrition LLC, USA); (2) NO_3_^−^-rich beetroot juice (BR: ~12 mmol of NO_3_^−^ total, Beet it; James White Drinks, Ipswich, UK) with two empty gelatin capsules; and (3) BR with two capsules containing pomegranate powder (POM: 1000 mg, Bulk Supplements, Hard Eight Nutrition LLC, USA); (23) (BR + POM), with each condition separated by a minimum of a 5 day washout period. On experimental days, participants consumed 2 × 70 mL of their allocated beverage and capsules 2.5 h before exercise given that peak plasma [NO_2_^−^] occurs ~2- to 3 h following NO_3_^−^ ingestion (34). After the experimental visits were completed, and on a separate laboratory visit, participants ingested two capsules containing 1,000 mg of POM 2.5 h prior to a resting blood draw for the determination of plasma [NO_3_^−^] and [NO_2_^−^] following the ingestion of POM alone. At the start of each experimental visit, we assessed the effectiveness of the blinding procedures by verbally asking whether the participants noticed any differences in the supplements ingested.

### 2.5. Measurements

#### 2.5.1. Plasma [NO_3_^−^] and [NO_2_^−^] analysis

A resting venous blood sample was obtained from the antecubital vein of the forearm by a trained member of the research team upon arrival to the laboratory for the assessment of plasma [NO_3_^−^] and [NO_2_^−^]. Samples were drawn into 6 mL lithium heparin tubes (Vacutainer, Becton-Dickinson, New Jersey, USA) and centrifuged at 3100× *g* at 4°C for 10 min within 2 min of collection. Plasma was extracted and stored in a −80°C freezer for the later analysis of plasma [NO_3_^−^] and [NO_2_^−^] using gas phase chemiluminescence as previously described (11). All glassware, utensils and surfaces were rinsed with deionized water to remove NO prior to analysis. Plasma samples were thawed then deproteinized using ice-cold ethanol precipitation prior to [NO_2_^−^] analysis. Specifically, samples were centrifuged at 14000× *g* for 10 min, and 200 μL of the supernatant was treated with 400 μL of ice-cold ethanol. Samples were then incubated on ice for 30 min, and subsequently centrifuged at 14000× *g* for 10 min. The [NO_2_^−^] of deproteinized plasma was determined by its reduction to NO using glacial acetic acid and aqueous sodium iodide and calibrated using sodium NO_2_^−^ standards. Following this, the deproteinized plasma samples were diluted prior to [NO_3_^−^] analysis such that 100 μL of the supernatant was added to 400 μL of deionized water. The [NO_3_^−^] of diluted deproteinized plasma was determined by its reduction to NO using vanadium chloride and hydrochloric acid and calibrated using sodium NO_3_^−^ standards. Supplements were diluted with deionized water and analyzed for [NO_3_^−^] and [NO_2_^−^] using the same methods employed for measuring plasma [NO_3_^−^] and [NO_2_^−^] and converted into mmol per 70 mL.

#### 2.5.2. Mood

The Brunel Mood Scale (BRUMS) (35, 36) is used to assess mood states in adult populations and was conducted prior to exercise as mood may have a mediating effect on resistance training performance (37). Using the standard response time frame of “How do you feel right now?,” 24 items representing six subscales (i.e., anger, confusion, depression, fatigue, tension, vigour; four-items per subscale) were captured using a five-point Likert scale (i.e., 0 = not at all, 1 = a little, 2 = moderately, 3 = quite a bit, 4 = extremely). Respective items were summed so that each subscale score ranged from 0–16 raw points. In general, elevated vigour and decreased anger, confusion, depression, fatigue, and tension subscale scores are viewed as positive outcomes.

#### 2.5.3. Vertical countermovement jumps

The vertical countermovement jumps were used to assess body mass, ballistic neuromuscular performance (e.g., power, velocity, height) of the lower-body extensors. Participants stood on an Advanced Mechanical Technology, Inc. (AMTI; Watertown, MA, USA) AccuPower-Optimized multi-axis force platform and were asked to jump as far upward as possible. As previously described (38), participants were tasked with executing a downward movement until the knees were flexed to approximately 90° and then maximally and explosively jumping upward while keeping their hands on their hips at all times. Participants were instructed to not flex their knees during the flight phase, to soften their impact with their feet at landing, and to give maximum explosive efforts. Following a standardized warm-up, participants performed 5 repetitions of the vertical countermovement jump with 1 min of rest between each repetition (38). Data were processed via AccuPower software, version 4.0 (AccuPower Solutions, Dickinson, ND, USA). During the 1 set x 5 repetitions of vertical countermovement jump, peak power, jump height, and peak velocity were recorded as was the five-repetition *average* propulsion mean force. For each repetition, the propulsion mean force represented the sum of all vertical force values divided by *N* number of data points, with *N* being the number of samples between zero velocity and take-off (39). The average propulsion mean force and peak power values were both normalized to two-thirds body mass (40).

#### 2.5.4. Kneeling countermovement push-ups

The kneeling countermovement push-up (41) was used to measure partial body weight, ballistic neuromuscular performance (e.g., force, flight time, velocity, propulsion) of the upper-body extensors. To maintain assessment consistency across participants and to limit strength-related constraints, all participants completed the push-up in the kneeling position with weightlifting gloves. While in a kneeling, elevated push-up position (arms extended and shoulder length apart, knees together and in contact with two stacked 2.5-inch foam pads [Fitness Maniac LLC, X-Large Balance Pad, Fort Worth, TX] so that the knees were in-line with the above ground force plate), participants were asked to descend by flexing their elbows to an angle of approximately 90° and then to immediately propel their upper body as high as possible (i.e., flight phase). This assessment was performed using an AMTI AccuPower-Optimized multi-axis force platform. Participants were instructed to not flex their elbows during the flight phase, to soften their impact with their hands at landing, and to give maximum explosive efforts. Following a standardized warm-up, participants performed 5 repetitions of the kneeling countermovement push-up with 1 min of rest between each repetition. Data were processed via AccuPower software, version 4.0 (AccuPower Solutions, Dickinson, ND, USA). During the 1 set x 5 repetitions of kneeling countermovement push-ups, peak force, flight time, and peak velocity were recorded as was the five-repetition *average* propulsion mean force (as previously described for the countermovement jump). Average propulsion mean force as well as peak force values were both normalized to two-thirds body mass (40) using the force data applied to the force plate by the hands (i.e., did not include the force applied at the knees which were off the force plate). Due to not having two synchronized fore plates to capture hand and knee force data, we investigated metrics such as flight time and peak force and did not (for example) calculate peak power for the push-up task per the recommendations of Dhahbi and colleagues (42).

#### 2.5.5. Back squats

Power and velocity measurements were obtained during back squats using a portable, wireless, commercially available, linear position transducer (GymAware, Kinetic Performance Technology, Mitchell, Australia), which has been previously used (13) and validated for test–retest reliability (43). During the 2 sets x 2 repetitions at 70%1RM, power and velocity were averaged across sets for the determination of mean power and mean velocity, and the highest power and velocity values were recorded for the determination of peak power and peak velocity.

#### 2.5.6. Force plate

All dynamic force-based metrics derived from a platform (i.e., power, jump height, force, flight time, velocity, propulsion) were obtained using an AMTI AccuPower-Optimized multi-axis portable force plate (Watertown, MA, USA) and AccuPower software version 4.0 (AccuPower Solutions, Dickinson, ND, USA) for the vertical countermovement jump and kneeling countermovement push-up at a sampling rate of 1,200 Hz. All vertical force data were left unfiltered to maintain the integrity of the raw data and because noise was not evident (44, 45). AccuPower is a gold-standard jumping and power analysis software.

#### 2.5.7. Linear transducer

A linear position transducer (GymAware, Kinetic Performance Technology, Mitchell, Australia) was attached to the barbell to assess metrics such as power and velocity of movement of the back squat.

### 2.6. Statistical analyses

One-way repeated-measures ANOVAs were used to investigate statistical differences in plasma [NO_3_^−^] and [NO_2_^−^], mood, and resistance exercise performance between conditions (PL vs. BR vs. BR + POM vs. POM alone). Significant main effects were explored *post hoc* and pair-wise using Fisher’s least significant difference tests which do not control family-wise error rates. Rather, all pair-wise *post hoc t*-tests were completed using the mean squared error (i.e., the experiment-wide error) of statistically significant ANOVAs (i.e., protected *t* tests). Pearson product–moment correlation coefficients were used to assess the significant relationships between changes in plasma [NO_2_^−^] and performance variables. Unless stated otherwise, requisite statistical assumptions were met prior to all inferential analyses (e.g., sphericity, normality of the residuals, extreme outliers). Effect sizes for ANOVAs were measured via partial eta-squared (*η_p_*^2^) in which small, medium, and large effects were operationalized as 0.01, 0.06, and 0.14, respectively (46). Effect sizes for *t*-tests were measured as Cohen’s *d_z_* in which small, medium, and large effects were operationalized as 0.2, 0.5, and 0.8, respectively (46, 47). Statistical significance was set to *p* ≤ 0.05 with all data presented as mean ± SD, unless otherwise stated. All data were analyzed using SPSS version 27 (IBM, Armonk NY).

## 3. Results

All participants reported consuming all servings of each supplement at the correct times and verbally confirmed that they had maintained their habitual exercise and dietary habits prior to each testing visit. Further, all participants verbally confirmed that they did not notice any differences between the supplements.

### 3.1. Supplement [NO_3_^−^]

The NO_3_^−^ concentration for PL, BR, and POM were ~ 0.05 mmol per 70 mL, ~5.96 mmol per 70 mL, and < 0.001 mmol per 1000 mg, respectively.

### 3.2. Plasma [NO_3_^−^] and [NO_2_^−^]

The CV% for duplicate samples was 1.5 ± 0.3% and 9.1 ± 10.3% for plasma [NO_3_^−^] and [NO_2_^−^], respectively. There was a main effect by condition on plasma [NO_3_^−^] (*p* < 0.001, *n_p_*^2^ = 0.88) and [NO_2_^−^] (*p* < 0.001, *n_p_*^2^ = 0.59) (Table 1). Plasma [NO_3_^−^] was higher in BR (*p* < 0.001, *d_z_* = 2.34) and BR + POM (*p* < 0.001, *d_z_* = 3.25) compared to PL. Plasma [NO_3_^−^] was higher in BR (*p* < 0.001, *d_z_* = 2.48) and BR + POM (*p* < 0.001, *d_z_* = 3.28) compared to POM. There were no differences between BR and BR + POM (*p* = 1.00) or PL and POM (*p* = 1.00). Plasma [NO_2_^−^] was higher in BR (*p* < 0.001, *d_z_* = 1.52) and BR + POM (*p* < 0.001, *d_z_* = 1.36) compared to PL. Plasma [NO_2_^−^] was higher in BR (*p* < 0.001, *d_z_* = 1.24) and BR + POM (*p* < 0.001, *d_z_* = 1.11) compared to POM. There were no differences between BR and BR + POM (*p* = 1.00) or PL and POM (*p* = 1.00). There were no significant correlations between the magnitude of increase in plasma [NO_2_^−^] and performance variables (*p* = 0.66).

**Table 1:** Indices of nitric oxide bioavailability following acute dietary nitrate and pomegranate powder supplementation.

Variable	PL		BR		BR + POM	
	Mean ± SD	95% CI	Mean ± SD	95% CI	Mean ± SD	95% CI
Plasma [NO_3_^−^] (μM)	62 ± 24	48.43–75.25	485 ± 172^*^	389.73–579.94	519 ± 135^*^	444.54–593.53
Plasma [NO_2_^−^] (nM)	186 ± 63	151.17–220.41	475 ± 213^*^	357.32–593.28	556 ± 294^*^	393.59–719.21

^*^Significant difference in BR and BR + POM compared to PL (*p* < 0.001). BR, nitrate-rich beetroot juice; beetroot; BR + POM, nitrate-rich beetroot juice and pomegranate powder; CI, confidence interval; NO_3_^−^, nitrate; NO_2_^−^, nitrite; POM, pomegranate powder; PL, nitrate-depleted beetroot juice.

### 3.3. Mood

Mood outcomes are displayed in Table 2.

**Table 2:** Summary of variations in mood across all experimental visits.

	PL		BR		BR + POM	
Variable	Average	Median	Average	Median	Average	Median
Anger	0.13 ± 0.35	0.00	0.53 ± 0.92	0.00	0.07 ± 0.26	0.00
Confusion	0.07 ± 0.26	0.00	0.13 ± 0.35	0.00	0.33 ± 1.05	0.00
Depression	0.27 ± 0.80	0.00	0.13 ± 0.52	0.00	0.27 ± 0.80	0.00
Fatigue	2.53 ± 2.33	3.00	2.13 ± 2.59	2.00	3.00 ± 3.32	2.00
Tension	0.73 ± 1.71	0.00	0.80 ± 1.78	0.00	0.47 ± 1.13	0.00
Vigor	7.33 ± 3.42	8.00	7.00 ± 3.68	7.00	7.33 ± 4.17	7.00

BR, nitrate-rich beetroot juice; BR + POM, nitrate-rich beetroot juice and pomegranate powder; PL, nitrate-depleted beetroot juice.

There was no effect of condition on anger (*p* = 0.09), confusion (*p* = 0.47), depression (*p* = 0.27), fatigue (*p* = 0.48), tension (*p =* 0.34), or vigour (*p* = 0.87).

### 3.4. Vertical countermovement jump performance

Neuromuscular performance outcomes during vertical countermovement jump are displayed in Table 3.

**Table 3:** Performance outcomes during a vertical countermovement jump exercise protocol for the determination of power, jump height, velocity, and propulsion following various combinations of acute supplementation with nitrate-rich beetroot juice, pomegranate powder, and nitrate-depleted beetroot juice.

Variable	PL		BR		BR + POM	
	Mean ± SD	95% CI	Mean ± SD	95% CI	Mean ± SD	95% CI
Peak Power (W/Kg^0.67^)	85.58 ± 10.05	80.12–91.15	88.50 ± 11.46^* a^	82.16–94.84	85.80 ± 10.14	80.19–91.41
Jump Height (cm)	42.61 ± 6.24	39.15–46.06	44.62 ± 6.96	40.77–48.48	42.60 ± 6.44	39.03–46.17
Peak Velocity (m/s)	2.88 ± 0.21	2.77–3.00	2.90 ± 0.25	2.77–3.04	2.92 ± 0.20	2.81–3.03
Average Propulsion Mean Force (N/Kg^0.67^)	27.02 ± 1.75	26.05–27.99	27.40 ± 1.65	26.49–28.32	27.16 ± 1.76	26.19–28.13

^*^Significant difference (*p* < 0.05); ^a^BR vs. BR + POM. BR, nitrate-rich beetroot juice; BR + POM, nitrate-rich beetroot juice and pomegranate powder; CI, confidence interval; cm, centimeters; m/s, meters per second; N/Kg^0.67^, newtons per 0.67 kilogram; PL, nitrate-depleted beetroot juice; W/Kg^0.67^, watts per 0.67 kilogram.

There was a main effect of condition (*p* = 0.04, *n_p_*^2^ = 0.20), with *post hoc* analyses revealing that peak power output increased in BR by ~3% compared to BR + POM (*p* = 0.009, *d_z_* = 0.78). While peak power output increased in BR by ~3% compared to PL, this did not reach significance (*p* = 0.07, *d_z_* = 0.50). There was no difference in peak power output between BR + POM and PL (*p* = 0.86). There was no main effect of condition on jump height (*p* = 0.08), peak velocity (*p* = 0.67) or average propulsion (*p* = 0.26).

### 3.5. Kneeling explosive push-up performance

Neuromuscular performance outcomes during kneeling explosive push-ups are displayed in Table 4. There was no effect of condition on peak force (*p* = 0.87), flight time (*p* = 0.59), peak velocity (*p* = 0.70), or average propulsion mean force (*p* = 0.94) during kneeling explosive push-ups.

**Table 4:** Performance outcomes during a kneeling explosive push-up exercise protocol for the determination of force, flight time, velocity, and propulsion following various combinations of acute supplementation with nitrate-rich beetroot juice, pomegranate powder, and nitrate-depleted beetroot juice.

Variable	PL		BR		BR + POM	
	Mean ± SD	95% CI	Mean ± SD	95% CI	Mean ± SD	95% CI
Peak Force (N/Kg^0.67^)	37.45 ± 7.96	33.04–41.86	36.99 ± 6.27	33.51–40.46	37.29 ± 6.96	33.44–41.15
Flight Time (s)	0.54 ± 0.19	0.43–0.64	0.53 ± 0.16	0.44–0.61	0.51 ± 0.14	0.43–0.59
Peak Velocity (m/s)	3.74 ± 0.82	3.29–4.20	3.86 ± 0.95	3.34–4.39	3.71 ± 0.51	3.43–4.00
Average Propulsion Mean Force (N/Kg^0.67^)	24.73 ± 2.82	23.17–26.29	24.83 ± 2.37	23.52–26.14	24.74 ± 2.14	23.55–25.92

BR, nitrate-rich beetroot juice; BR + POM, nitrate-rich beetroot juice and pomegranate powder; CI, confidence interval; m/s, meters per second; N/Kg^0.67^, newtons per 0.67 kilogram; PL, nitrate-depleted beetroot juice; s, seconds.

### 3.6. Back squat performance

Neuromuscular performance outcomes during back squats are displayed in Table 5. There was no effect of condition on peak power (*p* = 0.45), mean power (*p* = 0.72), peak velocity (*p* = 0.28), or mean velocity (*p* = 0.90) during back squats.

**Table 5:** Performance outcomes during a back squat exercise protocol for the determination of power and velocity following various combinations of acute supplementation with nitrate-rich beetroot juice, pomegranate powder, and nitrate-depleted beetroot juice.

Variable	PL		BR		BR + POM	
	Mean ± SD	95% CI	Mean ± SD	95% CI	Mean ± SD	95% CI
Peak Power (W)	1,461 ± 403	1237.91–1684.09	1,426 ± 426	1189.73–1661.60	1,409 ± 411	1181.57–1636.70
Mean Power (W)	605 ± 155	519.33–691.38	602 ± 137	526.09–677.57	597 ± 131	524.15–669.36
Peak Velocity (m/s)	1.42 ± 0.19	1.31–1.52	1.38 ± 0.15	1.29–1.46	1.39 ± 0.21	1.27–1.50
Mean Velocity (m/s)	0.74 ± 0.10	0.69–0.80	0.74 ± 0.06	0.71–0.78	0.74 ± 0.08	0.70–0.78

BR, nitrate-rich beetroot juice; BR + POM, nitrate-rich beetroot juice and pomegranate powder; CI, confidence interval; m/s, meters per second; PL, nitrate-depleted beetroot juice; W, watts.

## 4. Discussion

The main novel findings of the present study were that: (1) BR and BR + POM increased plasma [NO_3_^−^] and [NO_2_^−^] to a similar extent compared to PL; (2) peak power output improved during vertical countermovement jumps following BR compared to BR + POM; and (3) BR supplementation did not influence neuromuscular performance during vertical countermovement jumps, kneeling explosive push-ups or back squats compared to PL. These findings conflict with our hypotheses and suggest that neither independent NO_3_^−^ supplementation or co-ingestion of NO_3_^−^ with POM have effects on explosive power during resistance exercise.

### 4.1. The effect of co-ingesting beetroot juice and pomegranate powder on nitric oxide bioavailability

Both BR and BR + POM increased plasma [NO_3_^−^] and [NO_2_^−^] to a similar extent compared to PL. Increased plasma [NO_3_^−^] and [NO_2_^−^] after BR consumption in the current study is consistent with numerous previous reports of increased plasma [NO_3_^−^] and [NO_2_^−^] after acute BR ingestion, both prior to exercise (34, 48) and following a top up dose during exercise (49). Of the few previous studies to have examined the effects of co-ingesting NO_3_^−^ and antioxidant compounds, similar results have been obtained as those reported herein. For example, increases in plasma [NO_3_^−^] and [NO_2_^−^] were similar following the co-ingestion of NO_3_^−^ and N-acetylcysteine (34, 48), as well as NO_3_^−^ and ascorbic acid (50) compared to NO_3_^−^ supplementation alone. Given that POM has been previously shown to contain high antioxidant and polyphenolic content (22), in aggregate, current evidence indicates that the administration of NO_3_^−^ concomitant with antioxidant-based supplements may not have an appreciable impact on NO biomarkers, despite the potential for the antioxidant supplements, to preserve NO bioavailability by quenching ROS (19). Due to the limited available data, and that the present study did not measure antioxidant properties of POM, caution is required in the interpretation of data and further research is required.

POM has been investigated as a form of dietary NO_3_^−^ supplementation given that it was purported to contain NO_3_^−^ (23). However, the POM administered in the current study was devoid of nitrate such that plasma [NO_3_^−^] and [NO_2_^−^] were similar between PL and POM, and BR + POM and BR. From the limited available data, Crum et al. (25) found that 8 days of POM supplementation (~1.4–2.4 mmol of NO_3_^−^) significantly increased plasma [NO_3_^−^] compared to a placebo condition and lowered the oxygen cost of exercise. Although Crum et al. (25) reported a statistically significant increase in plasma [NO_3_^−^] following POM supplementation, the increase was only by ~12.9 μM, which is markedly lower compared to an increase of ~400 μM that we observed following acute BR ingestion (~12 mmol of NO_3_^−^). Moreover, from dose–response data, the minimum effective dose of ~8 mmol of NO_3_^−^ resulted in an increase of ~300 μM in plasma [NO_3_^−^] (34). The large difference in the magnitude of increase in plasma [NO_3_^−^] following POM compared to BR highlights the difference between supplements in their potential to impact NO bioavailability. Furthermore, an original contribution of the current study was that we analyzed [NO_3_^−^] in POM via the gold standard method of gas phase chemiluminescence. We found that POM contained <0.001 mmol of NO_3_^−^ per 1,000 mg. These results are much lower compared to Roelofs et al. (23) who reported that POM contained 109 ppm/1000 mg of NO_3_^−^ (~1.76 mmol of NO_3_^−^). Thus, our results challenge previous studies which suggested POM to be a rich source of NO_3_^−^ (23), especially since the minimum effective acute NO_3_^−^ dose is considered to be ≥8 mmol to elicit ergogenic effects (51). It is also possible that differences in [NO_3_^−^] in POM between the current and previous studies could be due to differences in supplement creation. In addition, we observed that POM ingestion did not alter plasma [NO_2_^−^] compared to PL, which corroborates our findings of negligible [NO_3_^−^] in POM in the current study. Together, these data indicate that POM contains a substantially lower amount of NO_3_^−^ in comparison to BR (~6 mmol of NO_3_^−^ per 70 mL) and may not be sufficient to markedly increase NO bioavailability to induce NO-mediated effects or work synergistically with BR. Therefore, although previous studies have found physiological and performance enhancing effects following POM supplementation (23, 25, 26), the extent to which these effects are linked to NO-related mechanisms is unclear, but likely to be small. A limitation of our study is that we did not implement an experimental condition for participants to ingest POM alone, and therefore we are not able to discern the independent effects of POM ingestion on the performance variables assessed in the current study.

### 4.2. The influence of NO_3_^−^ supplementation on resistance exercise performance

An original contribution of the current study was that we assessed power during vertical countermovement jumps. We observed that while an acute ~12 mmol NO_3_^−^ bolus, provided as BR, improved peak power during vertical countermovement jumps by ~3% compared to BR + POM (*d_z_* = 0.78), there was no significant difference between BR and PL (*d_z_* = 0.50). Our results indicate that there is potential for BR to improve explosive power output, at least when compared to BR + POM, which corroborates the findings of a recent meta-analysis reporting that dietary NO_3_^−^ can improve explosive power during dynamic movements, albeit with a small effect (4). The performance enhancing effects following dietary NO_3_^−^ ingestion have been attributed to improved contractile function, including a lower high-energy phosphate cost of force production (23, 25, 26), improved excitation-contraction coupling (6, 52), and more pronounced effects in type II muscle fibers (5, 6). These improvements might also account for similar previous observations in studies reporting NO_3_^−^ to enhance power output during cycling (53, 54), and knee extensions (18, 55, 56).

We observed that improved peak power following BR alone translated into a non-significant increase of 5% in jump height (*p* = 0.09, *d_z_* 0.48). Our results regarding the effects of NO_3_^−^ supplementation on jump height performance agree with some (14, 53) but not with other studies (15). For example, Jurado-Castro et al. (15) reported that an acute low dose of NO_3_^−^ (~6 mmol of NO_3_^−^) significantly improved countermovement jump height by ~6% in females, but in contrast, 6 days of NO_3_^−^ supplementation with a moderate dose (~12 mmol of NO_3_^−^ per day) had no effect on jump height in males (14). A possible explanation for the discrepancies between the present study and Jurado-Castro et al. (15) is that females may respond better to lower doses of NO_3_^−^ (29). Further research is required to understand the impact of dosing regimen on jump height performance and the extent to which sex-differences impact the ergogenic efficacy of NO_3_^−^.

We observed that combining BR with POM lowered peak power compared to BR during vertical countermovement jumps, which conflicts with our hypothesis. Although the mechanism for this effect is unclear, the model proposed by Reid et al. (20) posits that ROS regulates skeletal muscle contractile function in an inverted U pattern such that ROS levels below or beyond optimal concentrations may impair contractility. Therefore, co-ingesting NO_3_^−^ with another antioxidant may have consequently diminished any ergogenic potential via inducing a more reduced myocellular redox balance beyond what is optimal for contractile force production (57). However, the role of redox balance on contractile function is complex (19). For example, since NO is a reactive nitrogen species, the antioxidant and polyphenolic constituents from POM could either inhibit the canonical role of NO and/or reduce the production of peroxynitrite, thereby preserving NO, by attenuating the reaction between NO and superoxide, both of which could impact the activity of key contractile proteins such as sarcoplasmic reticulum calcium ATPase (1). It is interesting to note that, consistent with the results of the present study, previous studies that have co-ingested NO_3_^−^ with an antioxidant supplement did not provide additional benefits to exercise performance, above NO_3_^−^ supplementation alone (25, 58, 59). In addition to NO_3_^−^, BR contains antioxidant and polyphenolic constituents (60) and thus, it is possible that the ingestion of additional antioxidants alongside BR may modulate the redox balance to be unfavorable for exercise performance. It should be acknowledged that since we did not measure antioxidant and polyphenolic content of the beverages or blood redox biomarkers, a limitation of the current study is the lack of mechanistic insights into the impaired vertical jump peak power in BR + POM compared to BR. Therefore, caution is required for attributing the potential deleterious effects of POM to its antioxidant constituents. Future studies are encouraged to explore the effects of combining NO_3_^−^ with other antioxidant compounds to explore avenues for augmenting blood NO biomarkers and exercise performance with NO_3_^−^ supplementation.

Another original contribution of the current study was that we assessed the potential effects of NO_3_^−^ on explosive push-up performance. We observed that there was no influence of an acute dose of NO_3_^−^ on flight time, peak force, or propulsion during kneeling explosive push-ups. It has previously been suggested that NO_3_^−^ could be more efficacious in upper-body exercise given that upper body musculature could be comprised of a greater proportion of type II muscle fibers (61), and that NO_3_^−^ favorably influences type II muscle fibers. To date, few studies have examined the potential effects of NO_3_^−^ during upper-body resistance-type exercise, with conflicting results reported (9, 11, 13). For example, acute NO_3_^−^ ingestion improved the power and velocity of free-weights bench press by ~19% and ~ 6%, respectively (13), but in contrast, NO_3_^−^ has also been reported to have no effect on power during free weights bench press (11) and Smith-machine bench press (9). More studies are required to understand the impact of NO_3_^−^ on power output during various types of upper-body resistance exercise and explosive body weight outcomes.

NO_3_^−^ ingestion did not improve power output or velocity during back squats in the current study in agreement with some (9, 11) but not all studies (10, 15). For example, in males, an acute moderate NO_3_^−^ dose (~13 mmol NO_3_^−^) improved peak and mean power output during back squats by 15–22% (10) but other studies found no effect of NO_3_^−^ on power output during back squats after an acute low dose (~6 mmol NO_3_^−^) (9), acute moderate dose (~13 mmol NO_3_^−^) (11) or following 4 days of NO_3_^−^ ingestion (~13 mmol NO_3_^−^ per day) (11). A recent meta-analysis revealed that the efficacy of NO_3_^−^ on cycling performance may be better with higher NO_3_^−^ doses when provided acutely (62), but no study has examined how high NO_3_^−^ doses (≥ 16 mmol of NO_3_^−^) impact resistance exercise performance outcomes. Indeed, a high NO_3_^−^ bolus might further increase NO bioavailability, which has implications for improving performance (17); however, it was recently reported that higher doses worsened power output in older individuals and further research is required in young healthy adults (63). Another possible explanation is that NO_3_^−^ may be more efficacious during resistance exercise of a lower exercise intensity and a higher velocity of contraction. For example, in females, power output and velocity were improved following NO_3_^−^ ingestion during back squats performed at 50% 1RM but not at 75% 1RM (15). Furthermore, NO_3_^−^ increased the number of repetitions-to-failure at 60% 1RM and 70% 1RM but not 80% 1RM during back squats (9). In other exercise modalities, NO_3_^−^ supplementation elicited beneficial physiological and performance effects during higher pedaling rates compared to lower pedaling rates (64), and higher contraction velocities during isokinetic dynamometry (55), supporting the notion that the efficacy of NO_3_^−^ may be influenced by the velocity at which the contractions are completed. However, a recent study did not observe improved knee extensor power output regardless of velocity of contraction (14). Given these conflicting results, we cannot exclude the possibility that a lower intensity, and thus a faster velocity of contraction, could have increased the likelihood of an ergogenic effect during back squats. Thus, it may be important for future studies to standardize the tempo of resistance exercise movements.

## 5. Conclusion

The POM administered in the current study contained negligible NO_3_^−^ and POM ingestion did not increase plasma [NO_3_^−^] and [NO_2_^−^] compared to PL. Co-ingesting BR with POM did not alter the increase in plasma [NO_3_^−^] and [NO_2_^−^] compared to BR alone, but co-ingestion of BR with POM compromised peak power output during countermovement jumps when compared to BR ingested independently. However, compared to PL, BR ingestion did not alter performance in vertical countermovement jumps, explosive push-ups, or back squats. Therefore, based on the findings from the current study, acute co-ingestion of BR and POM to enhance resistance performance is not advised and acute BR ingestion was largely ineffective at increasing performance in the resistance testing battery administered in the current study. Future research is required to elucidate whether NO_3_^−^ can enhance resistance exercise performance and to elucidate the exercise settings and the NO_3_^−^ supplementation strategies that increase the ergogenic potential of NO_3_^−^ supplementation for resistance exercise.

## Data availability statement

The raw data supporting the conclusions of this article will be made available by the authors, without undue reservation.

## Ethics statement

The studies involving human participants were reviewed and approved by Pepperdine University Institutional Review Board. The patients/participants provided their written informed consent to participate in this study.

## Author contributions

RT, KMP LEW, IGL, STK, JPS, KKP, DWH, and IT performed data collection and acquisition. RT, KMP, LEW, IGL, STK, JPS, KKP, DWH, and IT organized the database. RT, KMP, LEW, IGL, JPS, KKP, DWH, and IT performed data analysis. RT performed the statistical analysis. RT, AP, and SB interpreted the data. RT, KMP, KKP, and AP wrote the first draft of the manuscript. RT, KMP, LEW, IGL, STK, JPS, DWH, IT, and AP wrote sections of the manuscript. All authors contributed to the article and approved the submitted version.

## Conflict of interest

The authors declare that the research was conducted in the absence of any commercial or financial relationships that could be construed as a potential conflict of interest.

## Publisher’s note

All claims expressed in this article are solely those of the authors and do not necessarily represent those of their affiliated organizations, or those of the publisher, the editors and the reviewers. Any product that may be evaluated in this article, or claim that may be made by its manufacturer, is not guaranteed or endorsed by the publisher.

## Footnotes

^1^ random.org
